# X Chromosome Inactivation Timing is Not e*XACT*: Implications for Autism Spectrum Disorders

**DOI:** 10.3389/fgene.2022.864848

**Published:** 2022-03-09

**Authors:** Janine M. LaSalle

**Affiliations:** Department of Medical Microbiology and Immunology, Perinatal Origins of Disparities Center, MIND Institute, Genome Center, Environmental Health Sciences Center, University of California, Davis, Davis, CA, United States

**Keywords:** DNA methylation, X chromosome inactivation, peri-implantation development, autism, neurodevelomental disorders

## Abstract

The etiology of autism spectrum disorders (ASD) is complex, involving different combinations of genetic and environmental factors. My lab’s approach has been to investigate DNA methylation as a tractable genome-wide modification at the interface of these complex interactions, reflecting past and future events in the molecular pathogenesis of ASD. Since X-linked genes were enriched in DNA methylation differences discovered from cord blood from newborns later diagnosed with ASD, this has prompted me to review and revisit the recent advancements in the field of X chromosome inactivation (XCI), particularly in humans and other primates. In this Perspective, I compare XCI mechanisms in different mammalian species, including the finding of the noncoding transcript *XACT* associated with X chromosome erosion in human pluripotent stem cells and recent findings from non-human primate post-implantation embryos. I focus on the experimentally challenging peri- and post-implantation stages of human development when the timing of XCI is prolonged and imprecise in humans. Collectively, this research has raised some important unanswered questions involving biased sex ratios in human births and the male bias in the incidence of ASD.

## Introduction

Autism spectrum disorders (ASD) is a collective term representing an etiologically diverse group of neurodevelopmental disorders characterized by deficits in social behaviors, deficits in language, and a gain of restrictive interests and repetitive behaviors (Lord et al., 2018). ASDs have increased in prevalence over the past several decades to their current prevalence estimate of 1 in 44 children (Mj et al., 2021). While major advances have been made in understanding genetics of ASD in rare syndromic cases, no single gene mutation of copy number variant can explain more than 1% of ASD cases (Geschwind, 2011; Rylaarsdam and Guemez-Gamboa, 2019). Instead, exome sequencing and association studies have identified hundreds of ASD candidate genes, which function at both the neuronal synapse and nucleus (Lasalle JM., 2013; Ayhan and Konopka, 2019). These autism candidate genes are collectively enriched for functions in chromatin regulation of gene expression and localization on the X chromosome (Mordaunt et al., 2020).

There is a strong male bias for ASD (4:1 male to female) that is poorly understood (Werling and Geschwind, 2013). In addition to genetics, *in utero* exposure to a multitude of maternal exposures, including air pollution, pesticides, Rubella infection, and obesity increase risk for ASD in the offspring (Lasalle J. M., 2013; Bolte et al., 2019). Such *in utero* exposures are preferentially harmful to male fetuses (Inkster et al., 2021). Epigenetic mechanisms that regulate gene expression through changes to chromatin and DNA methylation act at the interface of genetic and environmental factors for ASD risk and sex differences in ASD. While the epigenetics of ASD has been reviewed recently (Bastaki et al., 2020; Saxena et al., 2020; Mossink et al., 2021; Panisi et al., 2021; Reichard and Zimmer-Bensch, 2021; Rosenfeld, 2021), this Perspective will focus on the X chromosome in the epigenetic and genetic etiology of ASD and the sex bias in ASD. Specifically, to better understand our recent genome-wide discovery that ASD-associated DNA methylation differences in cord blood DNA were enriched on the X chromosome and included the primate-specific *XACT* regulatory RNA (Vallot et al., 2013; Mordaunt et al., 2020), I will review and discuss the latest findings on X chromosome inactivation, erosion, and escape from studies in human and other primates. By revisiting the question of why there is a sex bias in ASD through the lens of sex chromosome biology, I raise questions about the timing of epigenetic alterations to the X chromosome in pregnancies resulting in ASD and propose future directions to explore.

### The Why, When, and How of X Chromosome Inactivation

Because females have two X chromosomes compared to only one in males, X chromosome inactivation (XCI) is predicted to reduce the level of X linked transcripts in females as a mechanism of dosage compensation (Sahakyan et al., 2018; Heard and Rougeulle, 2021). In eutherian mammals, the implantation of the fetal trophoblast into the maternal decidua of the uterus is a critical trigger for the events of XCI. The result of XCI is the random inactivation of parental alleles, meaning that in each cell, either the maternal or paternal X chromosome becomes the inactive Barr body. Not all genes on the inactive X chromosome are subject to XCI, however. In human, between 8–16% of genes are predicted to escape XCI entirely, compared to 3–7% of X linked genes in mouse (Carrel and Willard, 2005; Cotton et al., 2013; Balaton et al., 2015). In human, another 10–32% of X-linked genes are subject to escape from XCI in certain tissues or individuals, leading to inherent variability in the dosage of X-linked genes in human females.

The mechanism of XCI is dependent on expression of the noncoding transcript *XIST*. *XIST* acts in *cis* by localizing to and coating the inactive X chromosome and recruiting polycomb repressive chromatin factors, resulting in a more compact and heterochromatic chromosome (Cerase et al., 2015; Loda and Heard, 2019). Once established, the XCI pattern is stably maintained for each daughter cell during the lifetime of a mammal in female somatic tissues. In contrast, because XCI patterns need to be re-established every generation, the primordial germ cells (PGCs) in embryonic life that are the precursors of oocytes or spermatogonia in adulthood must reactivate or maintain an active X chromosome. Different epigenetic mechanisms appear to be responsible for dosage compensation versus true XCI heterochromatinization depending on the stage and cell type, but the overall effect is the balancing of X linked gene expression between males and females. For individual somatic cells within females, this means that in each cell, X linked genes are monoallelically expressed from either the maternal or paternal chromosome.

### Interspecies Differences Within Mammals in the Developmental Timing of XCI

The precise timing of the cellular choice of which X chromosome gets inactivated in early embryonic development is importantly different across different mammalian species. Since mouse has served as the primary model of human developmental biology and genetics, most of the knowledge of XCI establishment comes from this rodent. In mouse, *Xist* is paternally imprinted in its expression pattern soon after fertilization and zygotic genome activation, so that the preimplantation female embryo already exhibits dosage compensation in X-linked expression patterns that are comparable to male embryos (Sahakyan et al., 2018). This imprinted pattern of XCI is maintained in the trophectoderm, but reversed in the inner cell mass of the blastocyst, as a transient period of *Xist* expression from both alleles, followed by the establishment of random XCI (Loda and Heard, 2019; Heard and Rougeulle, 2021). In mouse, the antisense transcript to *Xist*, named *Tsix*, determines the active allele (Stavropoulos et al., 2001).

Adjacent to *Xist*, and in the same transcriptional orientation as *Tsix*, is a different noncoding RNA called *Jpx*. *Jpx* binds to the chromatin loop factor CTCF and activates *Xist* by displacing CTCF from an inhibitory loop (Sun et al., 2013). While *Jpx* was initially described as an *Xist* activator in mouse, more recently it was demonstrated to also activate many developmental genes on autosomes through a similar mechanism of CTCF displacement (Oh et al., 2021). Consistent with *Jpx* escaping XCI, female mouse ES cells express twice the expression level as males (Sun et al., 2013), but whether that means that autosomal *Jpx* developmental gene targets are expressed at a higher level in female versus male preimplantation embryos was not determined in this study. Another recently described activator of *Xist* in XX mouse ES cells is an enhancer localized lncRNA called *Xert* (Gjaltema et al., 2021). Unlike *Jpx*, whose expression is ubiquitous in somatic tissues, the expression of *Xert* appears limited to the mouse epiblast at embryonic days 5.5–6.5 (Gjaltema et al., 2021), but expected sex differences in embryonic *Xert* expression were not directly determined in this study.

The main differences between XCI in mouse versus human are in the role of imprinting, the timing of XCI events, and the noncoding genes required. Imprinted expression of *Xist* is observed in marsupials throughout life, with the paternal X specifically inactivated, but mouse appears to be an outlier among eutherian (placental) mammals in its imprinted pattern of XCI pre-implantation (Sahakyan et al., 2018). In humans, the requirement of maintaining dosage compensation through XCI in the preimplantation embryo appears to be less critical, as human embryos and human female pluripotent stem cells in culture express *XIST* on both X chromosomes, together with biallelic transcription chromosome-wide (Okamoto et al., 2021). *XIST* is also expressed from the only X chromosome in human male preimplantation embryos. Single cell sequencing analyses of pre-implantation embryos and pluripotent stem cells have shown a lower X to autosome ratio in transcript levels than expected for accurate dosage compensation in human (Vallot et al., 2017).

Genetic and epigenetic differences in the long noncoding RNAs and regulatory sequences within the X inactivation center (Xic) between rodents and primates are likely responsible for the timing and imprinting differences. Specifically, while a *Tsix* homolog exists in the human Xic, human *TSIX* does not have an apparent conserved function in repressing *XIST* on the active X chromosome (Migeon et al., 2001; Migeon et al., 2002). *JPX* is conserved and ubiquitously expressed in human tissues and *JPX* expression precedes that of *XIST* in preimplantation embryos, acting as a potential enhancer of *XIST* transcription (Patrat et al., 2020). Similar to *XIST*, *JPX* escapes XCI and shows female biased expression in human tissues (Balaton et al., 2015). Interestingly, a primate-specific X-linked lncRNA from outside the Xic was identified and named *XACT* that mapped to human Xq23 (Vallot et al., 2013). Like *XIST*, *XACT* accumulates on the X chromosome in a RNA cloud-like structure, but unlike *XIST*, its expression and localization is limited to the active X chromosome following XCI in human pluripotent stem cells (Vallot et al., 2017).

Over time in culture, human female pluripotent stem cells exhibit a phenomenon called “X chromosome erosion” characterized by a loss of *XIST* and a gain of *XACT* localization and a partial expression of genes from the previously established inactive X chromosome (Shen et al., 2008; Silva et al., 2008). *In vivo*, there is a strong burst of correlated expression of *XIST* and *XACT* expression in the 4-8 cell pre-implantation embryo, and both lncRNAs coat the two X chromosomes in female and single X chromosome in male embryos at these stages (Vallot et al., 2017). *XACT* is predicted to compete with *XIST*, based on a more diffuse chromosomal accumulation of *XIST* when colocalized to the same X chromosome as *XACT* and the appearance of *XACT* on eroded X chromosomes (Vallot et al., 2015; Vallot et al., 2017). However, a recent study has questioned this model, as deletion or knockdown of *XACT* did not result in changes to transcriptional patterns or *XIST* localization patterns in eroded pluripotent stem cells (Motosugi et al., 2021). Instead, deletion or re-expression of *XACT* using CRISPR deletion or activation resulted in changes to neuronal differentiation (Motosugi et al., 2021), a finding of potential interest to ASDs.

While human early post-implantation stages are intractable for experimental investigations, a recent single cell sequencing analyses of cynomolgus monkeys has revealed some striking findings about the timing of XCI in peri-implantation life in this nonhuman primate (Okamoto et al., 2021). Specifically, that *XIST* expression and repressive chromatin marks are temporally uncoupled from XCI and dosage compensation. Instead, an uncommitted state of chromatin marked by histone H2A Lys119 ubiquitylation (H2AK119Ub) and histone H3 Lys27 trimethylation (H3K27me3) and chromosome compaction lasted beyond the loss of *XIST* localization on the future active X chromosome. By embryonic days 13–16, roughly 4–7 days post-implantation, XCI as measured by monoallelic expression and X to autosomal expression ratio was complete (Okamoto et al., 2021). In contrast, in the trophectoderm that becomes the placenta, XCI was mostly completed soon after implantation at embryonic day 11 in cynomolgus monkeys (Okamoto et al., 2021). While these specific features of delayed XCI in the earliest post-implantation embryos are not possible to study in humans, this primate model is likely much more similar to human XCI than mouse models. Unfortunately, *XACT* expression or localization patterns were not examined in this study.

### Sex Ratio Studies and Sex Bias Human in ASD

Studies of early preimplantation embryos from *in vitro* fertilization (IVF) clinics have suggested that sex differences exist based on sex chromosome content in the timing of recognizable stages, with males showing faster cell division and progression to blastocyst stage than females, although some of the results have not been replicated (Patrat et al., 2020). Because these stages precede the production of sex hormones, genetic differences in sex chromosomal gene expression are predicted to explain these differences. *SRY*, the Y chromosome encoded, male-determining transcription factor, is predicted to explain the direct effect of increased proliferation in male embryos, although differences in the dosage of X linked genes between males and females is also implicated. These intriguing results support the idea that human and other primate peri-implantation embryos may have incorporated genetic differences between the sexes into the critical implantation stage of successful reproduction. This idea fits with the discrepancy between the human sex ratio at conception (SRC), which is equal (Orzack et al., 2015), and the sex ratio at birth (SRB), which is slightly but significantly skewed towards a higher rate of male births (Long et al., 2021). A recent population study of the entire Swedish and half the American population refuted hypotheses of seasonal or temperature variations but found multiple air and water pollutants associated with both lower and higher SRB (Long et al., 2021). These results therefore argued against a hypothesized sex-specific selection but confirmed and expanded the significance of environmental factors associated with a skew of sex ratio between conception and birth. A study that examined combined sex chromosome analyses in first trimester human fetuses from induced abortion, chorionic villus sampling, and amniocentesis saw evidence of female mortality bias as early as 2 weeks post-conception (Orzack et al., 2015), which is when XCI was completed in the cynomolgus monkey model (Okamoto et al., 2021). Perhaps male preimplantation embryos have a selective advantage at implantation due to their faster proliferation and progression. Alternatively, female embryos could be slightly more likely to be unsuccessful at implantation because of the plasticity inherent in X-linked chromatin and transcriptional variations resulting from incomplete XCI processes.

### Epigenetics of ASD—A Brief History

Beginning around the early 2000s, human genetics researchers became initially interested in epigenetic etiologies for ASDs based on the discovery of human syndrome disorders with features of autism that had genetic causes of epigenetic differences. This list included Rett syndrome, an X-linked dominant disorder affecting females which was discovered in 1999 to be caused by mutations in the X-linked gene *MECP2* (Amir et al., 1999). In Fragile X syndrome, caused by a CGG repeat in the promoter of X-linked gene *FMR1*, it was DNA methylation and chromatin changes of the *FMR1* repeated allele that was the mechanism for silencing of the repeated allele in males (Willemsen et al., 2011). Maternal duplication of 15q11.3-q13.3, the same region that when deleted cause the imprinted disorders Prader-Willi or Angelman syndromes, was identified as a frequent copy number variant in ASD (Dup15q syndrome) (Hogart et al., 2008). While the genetic cases served as proof of principle for the involvement of epigenetics in ASD, later studies from my lab and others identified DNA methylation changes in idiopathic ASD cases in *MECP2*, *FMR1*, and *UBE3A* (Jiang et al., 2004; Nagarajan et al., 2006; Nagarajan et al., 2008; Field et al., 2019). The initial hypothesis was that both X-linked and imprinted genes may be inherently more susceptible to transcriptional errors because of the erasure and re-establishment of epigenetic silencing marks every generation (Schanen, 2006).

Later, genome-wide advancements in genetic and epigenetic technologies allowed a broader look beyond candidate genes that furthered interest in epigenetic etiologies of ASD. Whole exome sequencing identified genes with *de novo* mutations in rare ASD cases affecting chromatin regulators (Michaelson et al., 2012; Yu et al., 2013). Gene expression analyses of ASD postmortem brain pinpointed the significance of immune response genes in ASD pathogenesis (Gupta et al., 2014; Iossifov et al., 2014; Parikshak et al., 2016). Whole methylome analyses revealed differences in ASD genes that were convergent between idiopathic and syndromic ASD (Rett and Dup15q syndrome), (Vogel Ciernia et al., 2020), as well as between environmental (PCB 95) and genetic (Dup15q) etiologies (Dunaway et al., 2016). With improved availability of human samples from ASD epidemiological cohorts and brain banks, as well as the improvements in genomic technologies over the past 2 decades, this is an exciting time to be investigating epigenetic etiologies of ASD to help explain the gap in knowledge that genetics alone cannot explain.

### X-Linked Epigenetics in ASD–Revisited

While detecting epigenetic differences in postmortem brain may be most relevant to understanding the most impacted tissue in the pathogenesis of ASD, these studies have inherent limitations. First, the epigenetic changes are observed many years after diagnosis and postmortem artifacts and other variables are difficult to control. Second, the number of postmortem samples is extremely limited. Lastly, there is no diagnostic translational relevance for predicting which children may develop ASD, which is not accurately diagnosed until 3 years of age. So, the field has sought to identify “surrogate” tissues from young children with ASD that may reflect some of the epigenetic changes observed in brain. Blood is the easiest to procure and thus has been the most studied, as well as buccal and saliva samples. But even these studies do not solve the problem of detecting epigenetic changes prior to the development of ASD.

Over the past decade, my lab has collaborated with epidemiologists involved in two human prospective studies of ASD to better understand the epigenetics of ASD. Markers of Autism Risk in Babies–Learning Early Signs (MARBLES) and Early Autism Risk Longitudinal Investigation (EARLI) are both longitudinal studies of pregnancies at enriched risk for ASD because of recruitment from mothers with at least one child diagnosed with ASD (Hertz-Picciotto et al., 2018). We have performed unbiased whole genome bisulfite sequencing analyses to identify differential methylated regions (DMRs) in both placenta and cord blood samples (Zhu et al., 2019; Mordaunt et al., 2020; Zhu et al., 2022). For both newborn sample types, we observed a significant overlap in the genes mapping to ASD associated DMRs with those identified in postmortem brain samples from multiple studies. But apparently unique to cord blood was the enrichment for ASD DMR linked genes to the X chromosome and early developmental pathways in both males and females (Mordaunt et al., 2020). Interestingly, all genes on the X chromosome, not just those mapping to ASD DMRs, were found to be enriched for brain and embryonic expression, as well as ASD genetic risk, compared to all genes in the genome. Unlike transcriptome data from the same cohort which showed transcript differences related to immune and chromatin functions (Mordaunt et al., 2019), the ASD cord blood DMRs showed greater enrichment for early developmental and neurodevelopmental than immune functions. In mapping the ASD DMRs to chromatin state maps across multiple tissues, ASD DMRs were enriched for regulatory regions defined by bivalent chromatin (H3K4me3 + H3K27me3) across multiple tissues in both sexes. But for the X-linked ASD DMRs, there was a divergence between males and females, with enrichment for bivalent and other functional chromatin states in females but only quiescent regions (no detectable histone modifications) in males. Together, these results suggested that the ASD DNA methylation differences we were seeing in cord blood were somehow related to epigenetic processes in early post-implantation life related to XCI.

The X-linked genes identified in this DNA methylation analysis of ASD cord blood also pointed to relevance for both ASD and XCI. ASD DMRs mapping to *XACT* were replicated in both sexes and both cohorts. We also replicated prior candidate-based findings for the syndromic ASD genes *MECP2* and *FMR1*. In addition, of the X-linked genes mapping to cohort-replicated ASD DMRs, many were also implicated in ASD at three different levels (genetic, epigenetic, and transcriptome) from other published studies (Mordaunt et al., 2020). Table 1 expands this list of genes “triple replicated” for ASD relevance by including their X chromosome position, described functions, XCI escape status, and SFARI ASD Gene status. The functions of these genes are varied, including regulators of transcription, signal transduction, and mitochondrial metabolism. Most of the ASD relevant genes in Table 1 are subject to XCI and there are no universal “escapees”, although several are discordant between tissues or have some variation between individuals (Balaton et al., 2015). If there is an XCI mediated mechanism behind the occurrence of the epigenetic changes in ASD, it is more likely to be on the genes subject to XCI, which brings us to the potential connection between *XACT* and X chromosome erosion.

**TABLE 1:** X-linked genes and their functions with triple-level multi-omic implication in ASD (G = genetic, E = epigenetic, T = transcriptome).

Gene name	Band	XCI status Balaton et al. (2015)	Y Homolog	Protein name and function	SFARI gene	G	E	T
*AR*	Xq12	Mostly subject	Y pseudo	Androgen receptor, a steroid-hormone activated transcription factor and transcriptional activator	Suggestive	Henningsson et al. (2009)	Shulha et al. (2012); Nardone et al. (2014); Vogel Ciernia et al. (2020)	Gupta et al. (2014)
*BCOR*	Xp11.4	Mostly subject	Y pseudo	BCL6 co-repressor, an interacting corepressor of BCL6, a POZ/zinc finger transcription repressor	—	Gilissen et al. (2014); Kochinke et al. (2016)	Sun et al. (2016); Nardone et al. (2014); Vogel Ciernia et al. (2020); Wong et al. (2019)	Gandal et al. (2018); Parikshak et al. (2016); Gilissen et al. (2014); Kochinke et al. (2016)
*CASK*	Xp11.4	Subject	Y pseudo	Calcium/calmodulin dependent serine protein kinase, a multidomain scaffolding protein with a role in synaptic transmembrane protein anchoring and ion channel trafficking	High Conf	Gilissen et al. (2014); Kochinke et al. (2016)	Nardone et al. (2014); Vogel Ciernia et al. (2020)	Gandal et al. (2018)
*CUL4B*	Xq24	Mostly subject, escape in brain	Y pseudo	Cullin 4B, an E3 ubiquitin ligase required for the proteolysis of several regulators of DNA replication including chromatin licensing and DNA replication factor 1 and cyclin E	—	Gilissen et al. (2014); Kochinke et al. (2016)	Nardone et al. (2014)	Gupta et al. (2014)
*IDS*	Xq28	Subject	Y pseudo	Iduronate 2-Sulfatase, an enzyme involved in the lysosomal degradation of heparan sulfate and dermatan sulfate	—	Gilissen et al. (2014); Kochinke et al. (2016)	Sun et al. (2016)	Gandal et al. (2018)
*IL1RAPL1*	Xp21.3-p21.2	Discordant	no	Interleukin-1 Receptor Accessory Protein-Like 1, a member of the interleukin 1 receptor family that plays a role in synapse formation and stabilization	Suggestive	Gilissen et al. (2014); Kochinke et al. (2016)	Nardone et al. (2014); Sun et al. (2016); Vogel Ciernia et al. (2020)	Gandal et al. (2018); Gupta et al. (2014)
*MAOA*	Xp11.3	Mostly subject, escape in brain	no	Monoamine oxidase Type A, a mitochondrial enzyme that catalyzes the oxidative deamination of amines, such as dopamine, norepinephrine, and serotonin	Suggestive	Gilissen et al. (2014); Kochinke et al. (2016)	Nardone et al. (2014)	Parikshak et al. (2016)
*MAP7D3*	Xq26.3	Subject	no	MAP7 Domain-Containing Protein 3, a member of the mitochondrial associated protein 7 family that promotes the assembly and stability of microtubules	—	Autism Spectrum Disorders Working Group of The Psychiatric Genomics (2017)	Nardone et al. (2014); Sun et al. (2016)	Gandal et al. (2018); Parikshak et al. (2016)
*MECP2*	Xq28	Subject	Y pseudo	Methyl CpG-Binding Protein 2, a member of methyl binding proteins and a transcriptional regulator	Syndromic	Iossifov et al. (2014); Gilissen et al. (2014); Kochinke et al. (2016)	Sun et al. (2016); Vogel Ciernia et al. (2020)	Gandal et al. (2018)
*MID1*	Xp22	Mostly subject	Y pseudo	Midline 1, an E3 ubiquitin ligase member of the ‘RING-B box-coiled coil’ subgroup of RING finger proteins that localizes to microtubules	—	Gilissen et al. (2014); Kochinke et al. (2016)	Nardone et al. (2014); Shulha et al. (2012); Sun et al. (2016); Vogel Ciernia et al. (2020)	Gandal et al. (2018); Parikshak et al. (2016)
*PCSK1N*	Xp11.23	Subject	no	Proprotein convertase Subtilisin/Kexin Type 1 Inhibitor, an inhibitor of prohormone convertase 1, which regulates the proteolytic cleavage of neuroendocrine peptide precursors	—	Autism Spectrum Disorders Working Group of The Psychiatric Genomics (2017)	Nardone et al. (2014)	Gandal et al. (2018)
*PGK1*	Xq21.1	Subject	no	Phosphoglycerate kinase 1, a glycolytic enzyme that catalyzes the conversion of 1,3-diphosphoglycerate to 3-phosphoglycerate	—	Gilissen et al. (2014); Kochinke et al. (2016)	Nardone et al. (2014); Sun et al. (2016); Vogel Ciernia et al. (2020)	Tylee et al. (2017); Gandal et al. (2018)
*PTCHD1*	Xp22.11	Disocordant	Y pseudo	Patched Domain Containing 1, a hedgehog receptor required for development and function of the thalamic reticular nucleus, a part of the thalamus that is critical for thalamocortical transmission, generation of sleep rhythms, sensorimotor processing and attention	High Conf	Gilissen et al. (2014); Kochinke et al. (2016)	Sun et al. (2016); Vogel Ciernia et al. (2020)	Gandal et al. (2018); Gupta et al. (2014)
*SLC7A3*	Xq13.1	Mostly subject	no	Cationic Amino Acid Transporter 3, a transporter that mediates the uptake of the cationic amino acids arginine, lysine and ornithine in a sodium-independent manner	Suggestive	—	Nardone et al. (2014)	Mordaunt et al. (2019)
*SMS*	Xp22.11	Subject	no	Spermidine synthase, catalyzes the production of spermine from spermidine and decarboxylated S-adenosylmethionine	—	Gilissen et al. (2014); Kochinke et al. (2016)	Nardone et al. (2014)	Gupta et al. (2014)
*TBL1X*	Xp22.31-p22.2	Discordant	Y homolog	Transducin Beta Like 1 X-Linked, F-box-like protein involved in the recruitment of the ubiquitin/19S proteasome complex to nuclear receptor-regulated transcription units	Suggestive	Iossifov et al. (2014)	Nardone et al. (2014)	Gupta et al. (2014)

**FIGURE 1:**
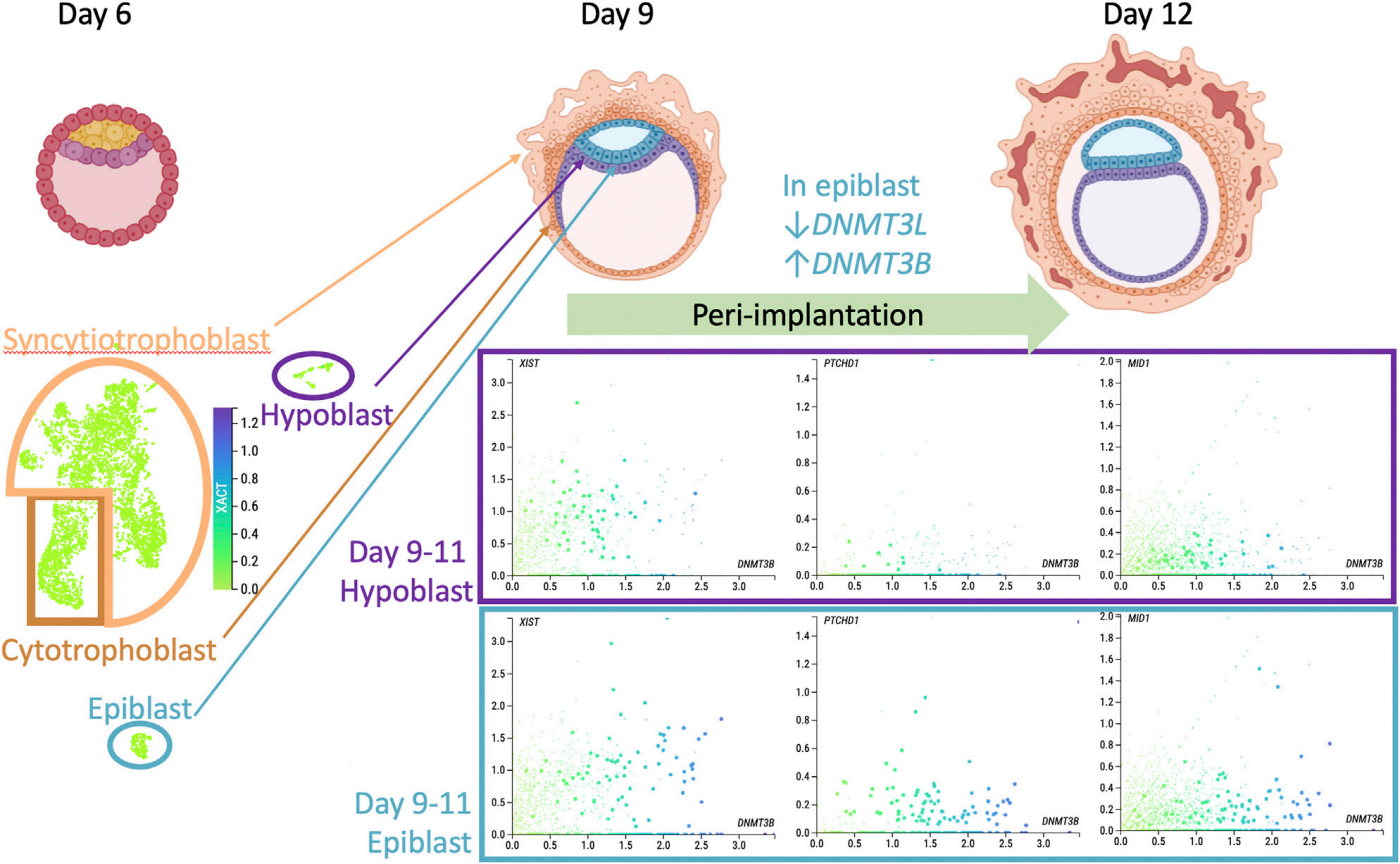
Could X chromosome erosion occur *in vivo*? Top row shows drawings of pre- (day 6 post fertilization) and peri-implantation human embryos with the major cell lineages labelled (from BioRender.com). Bottom panel presents data acquired from Mole et al., 2021 dataset in www.humanembryo.org which includes single cell RNA-seq from 16 human peri-implantation embryos, 8 at day 9 and 8 at day 11. While sex of embryos was not determined in the published study, a negligible level of *SRY* expression was observed, consistent with female sex. UMAP image on left bottom shows *XACT* expression at low uniform level across all cells and lineages. Scatterplots from cells from only the hypoblast (top) or epiblast (bottom) colored for level of *DNMT3B*, a marker of epiblast post-implantation differentiation (*x*-axis) compared to *y*-axis transcript levels: *XIST*, *PTCHD1*, *MID1* (two ASD relevant genes from Table 1). Scatterplots show the complexity of cellular variation in these X-linked transcripts, consistent with an extended period of XCI establishment and imprecise dosage compensation at the peri-implantation stage of human development.

X chromosome erosion has been described as an *in vitro* phenomenon unique to the culture of human pluripotent stem cells and not representative of the *in vivo* inner cell mass (ICM) (Bar et al., 2019). However, this conclusion was based on the comparison of human preimplantation (day 7) ICM bulk transcriptomes to individual pluripotent stem cell lines, and this raises the question that cellular variation may exist within individual ICM cells, particularly around implantation. In Figure 1, I revisit the question of whether the enrichment of X-linked genes we identified in ASD cord blood, a tissue derived from the epiblast lineage, could be related to their variable cellular expression and XCI status at a stage closer to post-implantation in humans. For this, the transcripts *XACT*, *XIST*, and two ASD relevant genes from Table 1, *PTCHD1* and *MID1*, were plotted from a recent single cell RNAseq data set from 16 human embryos cultured to 9 or 11 days post fertilization, bioinformatically sorted into epiblast, hypoblast, syncytiotrophoblast, and cytotrophoblast (www.humanembryo.org) (Mole et al., 2021). Interestingly, among the cell type markers of maturity in the epiblast identified in this study were transcriptional changes to two genes encoding DNA methyltransferases, specifically a decrease in *DNMT3L* and an increase in *DNMT3B* (25). *XACT* showed relatively low uniform expression throughout all lineages, unlike *XIST* expression (left scatterplots) which varied across cells and lineages and modestly correlated with *DNMT3B* expression. In contrast, the cells with the highest expression of *DNMT3B* epiblast (colored blue) showed lower expression of X-linked genes *PTCHD1* and *MID1* (from Table 1). These plots show that variability in X linked gene expression within cells of the peri-implantation human embryo exists and suggests caution in concluding the absence of X chromosome erosion based solely on bulk preimplantation ICM. Interestingly, *DNMT3B* deletion in pluripotent stem cells has the largest impact on the X chromosome, specifically in females at chromosomal domains marked by partial methylation (Martins-Taylor et al., 2012). *DNMT3B* deletion also results in premature neuronal differentiation, similarly as the effects of *XACT* deletion (Martins-Taylor et al., 2012). *DNMT3L* overexpression in a female neuroblastoma cell line led to increased DNA methylation over bivalent chromatin regions (Laufer et al., 2021), a chromatin state that predominates in the pre-implantation embryo (Voigt et al., 2013).

## Discussion

Our recent discovery that DNA methylation differences in cord blood from newborns later diagnosed with ASD were enriched for X-linked locations and early developmental functions has prompted me to revisit the recent X chromosome inactivation literature for insights into how and when these epigenetic errors may occur. *XACT* is particularly intriguing because it is primate-specific and appears to be involved in the phenomenon of X chromosome erosion *in vitro*, the prolonged delay of XCI that is observed in primates, and the timing of neuronal differentiation. This Perspective is therefore intended to raise new questions about this elusive stage of peri-implantation life in humans and if differentially DNA methylation patterns identified at birth may be reflecting gene by environment interactions in the etiology of ASD. First, is the sex bias in ASD somehow related to a higher likelihood that genetically susceptible female embryos may fail to successfully implant? Are human female peri-implantation embryos, because of their suspended state of poised chromatin and lack of absolute X-linked dosage compensation, epigenetically more plastic and therefore the sex more selectable by environmental variables, both positively and negatively? Can some of the features of X chromosome erosion occur *in vivo*, and if so, are these epigenetic changes a potential etiology of neurodevelopmental disorders? When and where do the X-linked DNA methylation differences observed in ASD cord blood occur: the developing brain, the pre- or post-implantation embryo, or the oocytes and/or sperm of the parents (or even their PGCs from the parents’ *in utero* life)? Clearly, these are all very difficult questions to answer because of the challenges in getting the right human samples. But new technologies such as single cell transcriptomes and epigenomes are expected to provide important new information. There is also a clear need for non-human primate studies in this area to better understand this critical life stage of early post-implantation for both XCI and ASD research.

## Data Availability

The original contributions presented in the study are included in the article/Supplementary Material, further inquiries can be directed to the corresponding author.
